# Serum Oxidative Stress Markers and Lipidomic Profile to Detect NASH Patients Responsive to an Antioxidant Treatment: A Pilot Study

**DOI:** 10.1155/2014/169216

**Published:** 2014-06-02

**Authors:** Paola Stiuso, Ilaria Scognamiglio, Marianna Murolo, Pasquale Ferranti, Carmela De Simone, Maria Rosaria Rizzo, Concetta Tuccillo, Michele Caraglia, Carmelina Loguercio, Alessandro Federico

**Affiliations:** ^1^Department of Biochemistry, Biophysics and General Pathology, Second University of Naples, Via De Crecchio 7, 80138 Naples, Italy; ^2^Department of Agriculture, University Federico II of Naples, Parco Gussone, Naples, 80055 Portici, Italy; ^3^Department of Medical, Surgical, Neurological, Metabolic and Geriatric Sciences, Second University of Naples, Piazza Miraglia 2, 80138 Naples, Italy; ^4^Department of Clinical and Experimental Medicine, Interuniversity Research Centre on Food, Nutrition and Gastrointestinal Tract (CIRANAD), Second University of Naples, via Pansini 5, 80131 Naples, Italy

## Abstract

Liver steatosis can evolve to steatohepatitis (NASH) through a series of biochemical steps related to oxidative stress in hepatocytes. Antioxidants, such as silybin, have been proposed as a treatment of patients with nonalcoholic fatty liver disease (NAFLD) and NASH. In this study, we evaluated, in patients with histologically documented NASH, the oxidant/antioxidant status and lipid “fingerprint” in the serum of NASH patients, both in basal conditions and after 12 months of treatment with silybin-based food integrator Realsil (RA). The oxidant/antioxidant status analysis showed the presence of a group of patients with higher basal severity of disease (NAS scores 4.67 ± 2.5) and a second group corresponding to borderline NASH (NAS scores = 3.8 ± 1.5). The chronic treatment with RA changed the NAS score in both groups that reached the statistical significance only in group 2, in which there was also a significant decrease of serum lipid peroxidation. The lipidomic profile showed a lipid composition similar to that of healthy subjects with a restoration of the values of free cholesterol, lysoPC, SM, and PC only in group 2 of patients after treatment with RA. *Conclusion.* These data suggest that lipidomic and/or oxidative status of serum from patients with NASH could be useful as prognostic markers of response to an antioxidant treatment.

## 1. Introduction

It is well known that nonalcoholic fatty liver disease (NAFLD) is manifested by a “metabolic” chronic liver damage due to an impaired “traffic” of lipids among adipose tissue, muscle, gut, and liver [1].

The occurrence of NAFLD is associated with numerous changes in the lipid composition of the liver [2] and the shift of these patients towards definitive steatohepatitis (NASH) is associated with changes in plasma lipidomic profile [3]. The clinical importance of NASH is related to its capacity to evolve in liver cirrhosis and cancer [4]. The principal risk factor for the development of NASH is insulin resistance [5–7] that increases lipolysis and releases free fatty acids (FFA) causing liver injury [8–10] by excessive liver lipid accumulation. Oversupply of free fatty acids induces an increase in mitochondrial H_2_O_2_ production that, in turn, oxidizes mitochondrial membranes and regulates activity of uncoupling protein 2 and carnitine palmitoyl transferase 1 [11]. Mitochondria play a key role in hepatocyte metabolism, being the site of *β*-oxidation and oxidative phosphorylation. Using a metabolomic approach, it has recently been shown that NASH is also characterized by decreased phosphatidylcholine (PC) and altered n3 and n6 polyunsaturated fatty acid (PUFA) metabolism [2, 3, 12]. Importantly, the levels of arachidonic acid (20:4 n6), the precursor of many biologically active eicosanoids, appear to be depleted [3]. It is not known if these changes can cause a variation in the circulating lipidome and if NASH can be, consequently, associated with a distinct lipidomic signature. Presently, there is no proven treatment for NASH and the introduction of drugs directly able to reduce oxidative stress, in association with lowering lipid accumulation, could be important in the control of these disorders. Silybin is a natural flavonoid and the main component of silymarin. Its derivative Realsil (RA) is a compound in which silybin is conjugated with phosphatidylcholine (PC) and vitamin E to enhance its intestinal absorption and its consequent bioavailability together with antioxidant and antifibrotic activity [13]. Silybin has a marked antioxidant activity both* in vitro *and* in vivo,* thus regulating glucose homeostasis in hepatocytes [13–15]. We have recently reported that a chronic treatment (for 12 months) with a dietary supplement of RA given orally twice a day significantly improves both liver damage plasma marker levels (AST, ALT, and *γ*GT) and liver histology in about 50% of patients with NAFLD and NASH [16]. In this study, we also observed that, despite the fact that no significant changes were observed in the global population for both dietetic regimen and body composition, in patients treated with RA, about 15% had a reduction of BMI values and 35% a reduction of blood glucose and HOMA test (marker of insulin resistance).

In this retrospective study, we addressed the effects of the chronic treatment with RA on both oxidative stress plasma markers and lipidomic profile in patients with NASH. Moreover, we have also evaluated the* in vitro* effects induced by sera from NASH patients on lipid accumulation in hepatoblastoma HepG2 cells.

## 2. Subjects and Methods

The study was performed after approval by the Ethic Committee according to Helsinki Declaration. The trial was registered with the European Clinical Trials Database (EudraCT, reference 2005-000860-24). We selected for our purpose frozen serum at −80° of 30 patients with histological documented NASH according to literature data [17] and treated for 12 consecutive months with Realsil (IBI-Lorenzini, Italy, RA) (active components: silybin 94 mg, phosphatidyl choline 194 mg, and vitamin E acetate 50% (*α*-tocopherol 30 mg) 89.28 mg) orally twice daily. Baseline clinical characteristics of the study population are summarized in Table 1. The histological diagnosis was established using H&E and Masson trichrome stains of formalin-fixed paraffin-embedded liver and graded in a blinded fashion according to the NAFLD scoring system proposed by the National Institute of Diabetes and Digestive and Kidney Diseases NASH Clinical Research Network. A NAFLD activity score (NAS) ≥5 corresponded to a diagnosis of “definitive NASH”, a score of 3-4 corresponded to “borderline NASH”, and a score of <3 corresponded to “simple steatosis” [16, 18].

### 2.1. Extraction of Serum Lipid and MALDI-TOF MS Analysis

Phospholipids were extracted in chloroform-methanol according to Bligh and Dye [19]. Methanol-chloroform (2 : 1 v/v; 800 *μ*L) was added to the serum (200 *μ*L). Phase separation is induced by adding 200 *μ*L of water. The mixture was centrifuged at 1000 g for 10 min. The upper phase was discarded and the lower chloroform phase was evaporated to dryness under a stream of nitrogen. The lipids were dissolved in 100 *μ*L of chloroform. A 2 *μ*L aliquot was used for MALDI-TOF MS determination. MALDI-TOF MS experiments were carried out by loading lipid mixtures (1 mL from a solution 0.02 mg/mL in H_2_O/0.1% v/v TFA) on the stainless steel target together with 1 *μ*L of matrix 2,5-dihydroxybenzoic acid (10 mg in 1 mL MetOH/0.1% v/v TFA). Spectra were acquired on a PerSeptive Biosystems (Framingham, MA, USA) Voyager DE-PRO mass spectrometer, equipped with a N_2_ laser (337 nm, 3 ns pulse width) operating either in linear or in reflector positive ion mode, using the delayed extraction technology. In the analysis of lipids, laser power was maintained at the lowest possible values in order to prevent insource fragmentation. To check repeatability, spectra were acquired in triplicate at least.

### 2.2. Thiobarbituric Acid-Reactive Species (TBARS) Levels

Samples were incubated with 0.5 mL of 20% acetic acid, pH 3.5, and 0.5 mL of 0.78% aqueous solution of thiobarbituric acid. After heating at 95°C for 45 minutes, the samples were centrifuged at 4000 r.p.m. for 5 minutes. In the supernatant fractions TBARS were quantified by spectrophotometry at 532 nm [20]. Results were expressed as TBARS *μ*M/*μ*g of serum protein. Each data point is the average of triplicate measurements, with each individual experiment performed in duplicate.

### 2.3. Nitrite Levels

NO is rapidly converted into the stable end products nitrite and nitrate. Nitrite was measured by the Griess reaction as reported in literature [21]. Briefly, 10 *μ*L of serum was mixed with an equal volume of Griess reagent (0.5% sulfanilamide, 2.5% H_3_PO_4_, and 0.05% naphthylethylene diamine in H_2_O) and incubated for 10 min at room temperature. Absorbance was assayed at 550 nm and compared with a standard curve obtained using sodium nitrite.

### 2.4. Catalase Activity

Catalase (CAT) activity was measured using Catalase Assay Kit (Cayman Chemical Ann Arbor, MI) according to the manufacturer's protocol. Each data point was performed in triplicate, and the results were reported as mean absorption ± standard deviation.

### 2.5. Superoxide Dismutase (SOD) Activity

Activity of superoxide dismutase (SOD) was measured with a superoxide dismutase assay kit (Cayman Chemical, Ann Arbor, MI) according to the manufacturer's protocol [22, 23]. Each data point was performed in triplicate, and the results were reported as mean absorption ± standard deviation.

### 2.6. Treatment of the HepG2 Cells with Sera from NASH Patients

The histological definition of steatosis is the visible accumulation of lipid droplets in more than 5% of hepatocytes. To determine if the serum of patients NASH may induce steatosis, HepG2 cells were cultured for 72 hours with pools sera of groups 1 and 2 of patients. We used both sera from T0 and T12 times. Oil Red O (ORO) methods were utilized for detecting intracellular lipids.

### 2.7. Statistical Analysis

Values are expressed as the mean ± SE. The significance of the difference between the control and each experimental test condition was analysed by unpaired Student's *t*-test, and *P* < 0.05 was considered statistically significant.

## 3. Results

### 3.1. Evaluation of Serum Oxidative Stress Markers and Metabolic Parameters in NASH Patients in Basal Conditions and after the Chronic Treatment with RA

Although we do not find any significant difference between NASH patients and controls as mean values, due to both high interindividual variability and sample size, the individual analysis of oxidative stress markers (TBARS and NO) and antioxidant enzyme activities (SOD and CAT) showed the presence of two distinct groups of patients. In the first group (group 1) of NASH patients (11/30), we found very low levels of TBARS if compared to those of healthy controls. In this group, the treatment with RA significantly (about 5-fold, *P* < 0.0001) increased mean serum levels of both TBARS and NO that overcame the mean values recorded in healthy subjects (*P* < 0.0001). The second group (group 2, 19/30) of NASH patients presented very high mean basal (T0) values of TBARS if compared to those of healthy subjects; in these patients the treatment with RA significantly decreased the TBARS mean values (2-fold, *P* < 0.0001), while NO mean values were almost unaffected by the pharmacological treatment (Table 2). In Table 2, we also reported both superoxide dismutase (SOD) and catalase (CAT) activity in the two previously defined groups before and after 12 months of RA treatment. In group 1 a significant decrease (*P* = 0.01) of mean values of CAT activity was found if compared to those of normal subjects, while in group 2 a significant increase of mean values of SOD activity (*P* = 0.01) was recorded. A separate system of scoring the features of NAFLD, called NAFLD activity score (NAS), was developed as a tool to measure changes in NAFLD during the therapeutic trials [18]. Interestingly, group 1 presented higher NAS scores (4.67 ± 2.5) when compared to that of group 2 (3.8 ± 1.5) indicating a higher basal severity of the disease. The chronic treatment with RA changed the NAS score in both groups (Table 3); in group 1 we observed a nonsignificant decrease (NAS = 3.6 ± 1.0, *P* = 0.057) while was significantly decreased in group 2 (NAS = 2.5 ± 0.51, *P* = 0.0058). NASH has been reported to be a component of the so-called “metabolic syndrome,” that is, a cluster of closely associated abnormalities related to the insulin-resistant phenotype [24]. In group 1 the mean BMI, insulin, and HOMA values did not significantly change after the treatment (*P* > 0.05), while mean glucose blood concentration significantly decreased (*P* = 0.05). On the other hand, in group 2 mean BMI (*P* = 0.005), insulin, and HOMA values significantly decreased (*P* = 0.001), while the concentration of glucose did not change after the pharmacological treatment.

### 3.2. Lipidomic Profile of Controls and NASH Patients before and after the Chronic Treatment with RA

**Figure 1:**
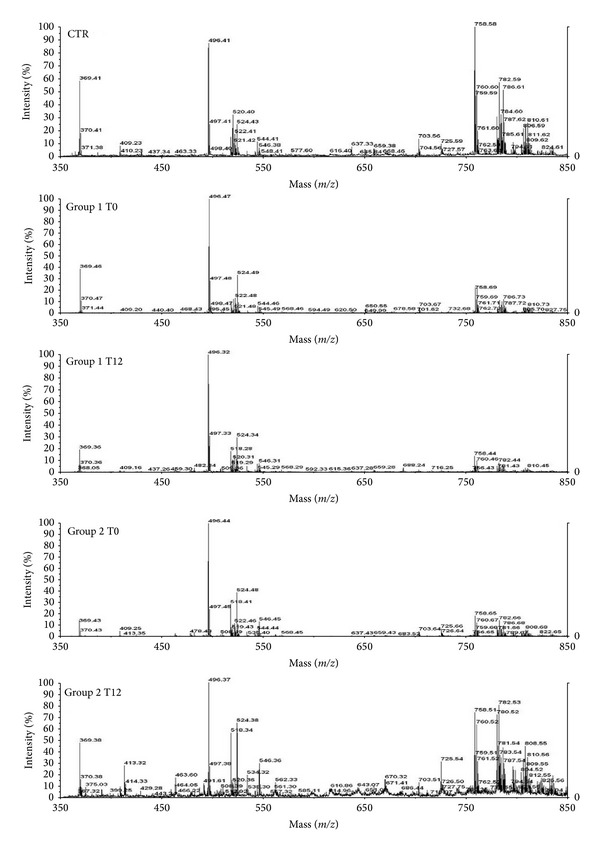
Positive ion MALDI-TOF MS mass spectra of choline phospholipid molecular species in lipid extracts from healthy individuals (CTR), group 1 and group 2 NASH patients at T0 and after 12 months of RA administration. Aliquots of chloroform extracts were analyzed directly by MALDI-TOF MS as described in Section 2.1 Selected peaks are indicated by their* m*/*z* values. For detailed peak assignments see Table 3.

**Figure 2:**
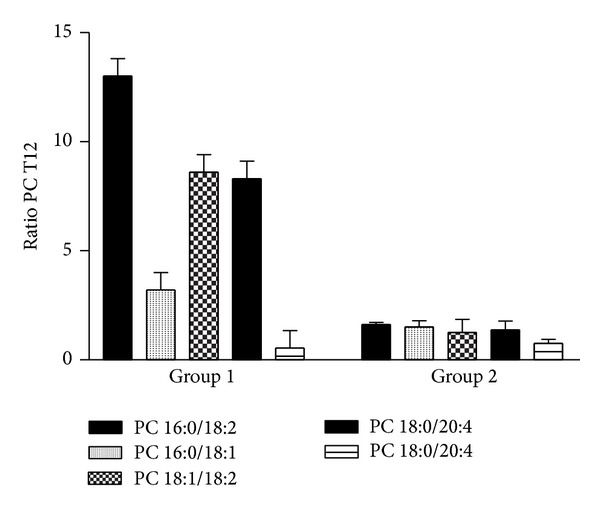
PC ratio of healthy subject and NASH patients after chronic administration of Realsil.

The sera of each group of subjects enrolled in the study (healthy subjects and groups 1 and 2 of patients) were separately pooled for the analysis of lipidomic profile by positive ion MALDI-TOF MS. In Figure 1, the positive ion MALDI-TOF mass spectra of the organic lipid extracts from sera of healthy subjects (CTR), group 1, and group 2 of NASH patients at T0 and after the chronic administration of RA (T12) are shown. The level of lysophosphatidylcholine (lysoPC) within palmitic acid (*m*/*z* = 496,36) was not significantly different among the three studied groups. However, the lipid species as free cholesterol, sphingomyelins (SM), and PCs in NASH patients at T0 (group 1 and group 2) were basally decreased if compared to those of healthy subjects. Only the NASH patients of group 2 after 12 months of RA treatment (T12) showed a lipid profile similar to that of healthy subjects with a restoration of the values of free cholesterol, lysoPC, SM, and PC. In Table 4 the identification and quantification of major plasma circulating lipids in healthy subjects, group 1, and group 2 at T0 and T12 are reported. The peaks with* m*/*z* between 520 and 524 were identified as LysoPC within linoleic (18:2), oleic (18:1), and stearic acid (18:0). In the healthy subjects, the three classes of lipids were uniformly represented, while both groups of NASH patients showed a major percentage of lysoPC within stearic acid (18:0) if compared to LysoPC 18:1 and 18:2, even after the treatment with RA. The ratio between PC percentage of healthy subject and that of NASH patients at T12 was reported in Figure 2. Only in Group 2 the ratio was about 1 after the chronic administration of RA. These findings may be due to an increase of both Δ 9 stearoyl-coA desaturase (SCD) and elongase activity. Importantly, in the NASH patients the levels of PC with arachidonic acid (Table 4), the precursors of many biologically active eicosanoids, were very low, but they increased only in group 2 at T12. RA treatment induced in both groups an increase of PC 18:0/20:3, with a partial restoration of their levels to those of healthy subjects.

### 3.3. *In Vitro* Effects of Serum from NASH Patients on Lipid Accumulation in HepG2 Cells

**Figure 3:**
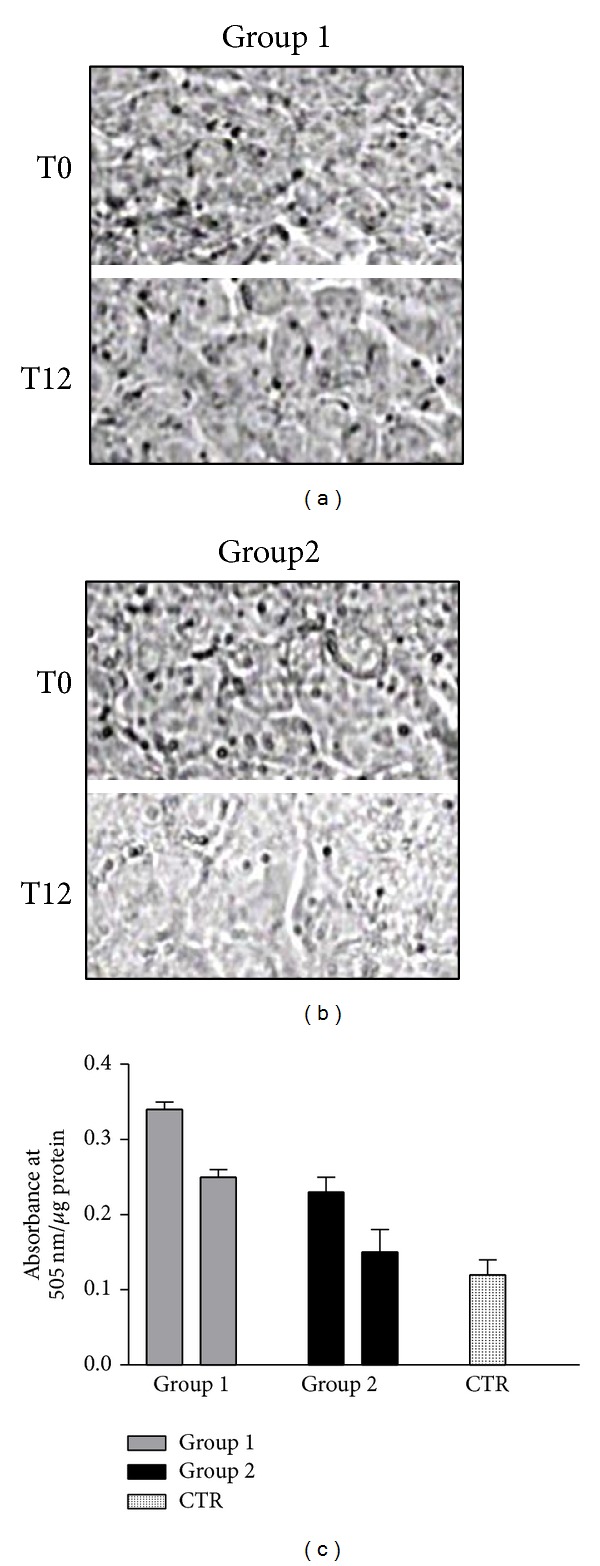
Serum NASH-induced steatosis in HepG2 cells determined by ORO staining ((a) and (b)) and ORO colorimetric assay (c). (a) The HepG2 cells were treated for 72 h with serum of group 1 at T0 and T12. (b) The HepG2 cells were treated with serum of group 2 at T0 and T12. (c) Oil red O colorimetric assay was determined on HepG2 cells after 72 h of incubation with NASH sera of groups 1 and 2 at T0 and T12.

The histological definition of steatosis is the visible accumulation of lipid droplets in more than 5% of hepatocytes. To determine if the serum of NASH patients may induce steatosis and if RA can be involved in lipid cell accumulation, HepG2 cells were cultured for 72 hours with pooled sera from group 1 or 2 at T0 and T12 or from healthy subjects. Oil Red O (ORO) method was used for the detection of intracellular lipids (Figure 3). ORO staining microscopy revealed lipid droplets accumulation in the cytoplasm of HepG2 cells after treatment with sera (groups 1 and 2) at T0 and a decrease of intracellular lipid only in the cells incubated with group 2 serum at T12 (Figures 3(a) and 3(b)). No changes were recorded in the cells exposed to the sera from healthy subjects (data not shown). In order to quantitatively assess lipid accumulation in HepG2 cells, we performed ORO colorimetric assay [25]. In Figure 3 panel (c), the quantitative ORO colorimetric assay on HepG2 cells after 72 h of incubation with NASH sera from groups 1 and 2 at T0 and T12 is shown. The effects of the sera of both groups 1 and 2 at T0 determined an about 2.5-fold increase of the lipid droplets if compared to those of untreated HepG2 cells (*P* < 0.001). The sera of group 2 NASH patients after treatment with RA (T12) induced an about 40% significant decrease of lipids accumulation if compared to that of HepG2 treated with the sera from T0 (*P* < 0.001).

## 4. Discussion

The diagnosis of NASH is defined by the presence of specific histological abnormalities determined at liver biopsy. Therefore, in all studies and trials on NAFLD, liver histology is the gold standard for the evaluation of response to treatments [17]. Serum markers of lipid peroxidation are generally used to evaluate the “oxidative stress” status* in vivo* in patients with NASH. The data of the present study suggest that, despite apparently similar clinical, biochemical, and histological characteristics that were found in all patients, two distinct groups of patients can be detected according to the modification of parameters of oxidative stress and lipid profiling. These two groups of patients have also a different sensitivity to the treatment with RA. Group 1 was characterized by lower lipid peroxidation as evaluated by TBARS assay, not due to increased SOD and CAT activity, while group 2 showed higher values of TBARS again with normal activity values of the scavenger enzymes. Insulin resistance (IR) was a common feature of both groups. Moreover, group 1 presented higher basal histological score (4.67 ± 0.5) corresponding to a greater severity of disease, while group 2 had a NAS score of 3.8 ± 0.6 corresponding to borderline NASH [17]. The excessive liver lipid accumulation in the pathogenesis of NASH can result from one or a combination of the following metabolic alterations: (i) decreased *β*-oxidation of fatty acids; (ii) increased fatty acid synthesis due to upregulation of lipogenic pathway; (iii) increased delivery of fatty acids from adipose and other organs due to lipolysis associated with peripheral insulin resistance (IR) and inhibition of VLDL-triglyceride [16].

Lower TBARS values in the serum of group 1 may be due to a greater hepatic intracellular accumulation of circulating FFA, mobilized by IR, which are not metabolized, as demonstrated by the significant 3-fold increase of ORO values of cells treated with group 1 T0 sera. In group 2 higher TBARS values could be correlated to the hepatocytes accumulation of circulating FFA, mobilized by IR, that are partially metabolized by *β*-oxidation with production of toxic aldehydes and their subsequent release into the circulation. Interestingly, low levels of serum TBARS were correlated to higher NAS score, while higher TBARS levels corresponding to NAS score were correlated to a milder disease. The chronic treatment with RA induced changes of serum oxidative status, metabolic parameters, and NAS score in both groups. In group 1, we observed an increase in TBARS values, presenting values higher than the control ones, a decrease of fasting glucose, a variation of NAS score that corresponded to borderline NASH (see Table 3), and a decrease of about 30% of ORO values compared to the T0 sera-treated cells. Instead, group 2 showed a significant decrease in TBARS value, BMI, insulin levels, HOMA test, and ORO values that resulted slightly higher than the control-treated cells, after RA treatment. These results demonstrate that 12 months of chronic administration of RA significantly improves group 2 disease as shown by NAS score variation from 3.6, that corresponded to “borderline NASH”, to 2.5 ± 0.51 corresponding to “simple steatosis.”

In the present study, we have also evaluated the effects of the treatment on serum “lipidomics” by MALDI-TOF mass spectrometry. More specifically, phospholipids are important components of all mammalian cells and have a variety of biological functions: (i) they form lipid bilayers that provide structural integrity necessary for protein function; (ii) they function as an energy reservoir (e.g., triglycerides); and (iii) they serve as precursors for various second messengers. In this light, lipid and phospholipid metabolism have an important role in the determination of NASH and the study of the modifications in the sera could reflect the lipidic metabolism in the liver [2, 3]. In fact, the study of the circulating “lipidome” does not provide direct information about changes in the liver but it is a tool to determine the effect of chronic treatment on whole-body lipid metabolism. We have found that lipid species as free cholesterol, SM, and PC in NASH patients at T0 (group 1 and group 2) were decreased compared to those of healthy subjects. In group 2 NASH patients, the chronic treatment with RA restored the levels of cholesterol and phospholipids to normal values. It is noteworthy that SMs are synthesized in the lumen of the Golgi apparatus [26] and move to the outer leaflet of the plasma membrane by vesicular membrane transport [27]. Moreover, SMs have high affinity for cholesterol and form a complex with cholesterol in the outer leaflet of the plasma membrane. RA treatment induces, only in group 2, the release of free cholesterol and SMs into the serum. It can be hypothesized that this release can be due to the increase of production of Lyso-PC 18:0 that is an amphipathic molecule and possesses “detergent-like” properties likely promoting the cholesterol-SM efflux [28]. These efflux-promotive effects of lyso-PC were confirmed by the fact that group 2 T12 sera-treated cells showed lower ORO staining than the basal cells (HepG2 treated with group 2 T0 sera). These results suggest that lyso-PC may inhibit the lipid accumulation in liver and the development of NASH disease or enhance its regression by stimulating cholesterol-SM efflux.

In conclusion, this is the first study, at least to our knowledge, that suggests that 12 months of treatment with RA can be useful in order to ameliorate the metabolic asset of patients affected by mild NASH. Finally, our findings suggest that the treatment of these patients with RA induces specific changes of lipidomic profile likely due to a different metabolic response of the patients that should be stratified also for other metabolic alterations (age, sex, AST, ALT, GGT levels, etc.). The understanding of the metabolic alterations at the basis of NASH could be useful in the future to have powerful predictive serum markers that can drive the clinicians in the treatment of this disease.

## Figures and Tables

**Table 1:** Main findings of patients with NASH and controls.

	NASH patients	Control subjects
Number	30	10
Age (yr)	40.8 ± 10.3	40 ± 12
Gender (M/F)	15/15	7/3
BMI (kg/m^2^)	29.9 ± 4.6	25.1 ± 2
Obesity	6/30	0
Diabetes mellitus	4/30	0
Hypercholesterolemia	4/30	0
Hypertriglyceridemia	3/30	0

**Table 2:** Serum TBARS, NO levels, and SOD and Catalase enzyme activities in two groups of NASH patients basal conditions (T0) and after 12 months of treatment with RA (T12).

Parameters	Group1		Group2		
	T0	T12	T0	T12	CTR
TBARS (*μ*M/*μ*g prot.)	0.0044 ± 0.0003	0.021 ± 0.001	0.074 ± 0.006	0.047 ± 0.004	0.01 ± 0.002
NO (nmol/*μ*g prot.)	0.011 ± 0.003	0.025 ± 0.003	0.135 ± 0.03	0.13 ± 0.027	0.0002 ± 0.00001
SOD activity (U/ng prot.)	0.121 ± 0.03	0.103 ± 0.02	0.159 ± 0.02	0.32 ± 0.056	0.15 ± 0.06
CAT activity (nmol/ng prot.)	1.3 ± 0.3	0.92 ± 0.08	1.5 ± 0.38	1.33 ± 0.185	1.5 ± 0.2

TBARS: thiobarbituric acid-reacting substances; NO: nitric oxide; SOD: superoxide dismutase; CAT: catalase.

**Table 3:** Metabolic data in two groups of NASH patients basal conditions (T0) and after 12 months of treatment with RA (T12).

Parameters	Group 1 T0	Group 1 T12	% variation	*P*	Group 2 T0	Group 2 T12	% variation	*P*
BMI	30 ± 1.86	30 ± 1.8	0	ns	28 ± 0.50	26 ± 0.50	9	0.005
Glucose	116 ± 10	105 ± 8.2	−10	0.05	99 ± 2.15	99 ± 2	0	ns
Insulin	18 ± 2.26	17 ± 3.77	−8	ns	23 ± 4.34	14 ± 1.9	−40	0.001
HOMA	5 ± 1	4.5 ± 1.0	−11	ns	5.97 ± 0.6	3.43 ± 0.5	−42	0.001
AST	40 ± 19	27 ± 7	−33	0.05	72 ± 31	41 ± 14	−42	0.01
ALT	40 ± 17	35 ± 10	−14	ns	72 ± 39	50 ± 9	−31	0.05
GGT	67 ± 31	43 ± 12	−35	0.05	101 ± 81	84 ± 75	−16	ns
Steatosis score	1.86 ± 0.90	1.8 ± 0.7	−0.01	ns	1.8 ± 0.8	1.2 ± 0.6	−33	0.01
NAS score	4.67 ± 1.5	3.6 ± 1.15	−29	ns	3.8 ± 1.5	2.5 ± 0.51	−70	0.001
Portal infiltration	1.33 ± 0.8	1.0 ± 0.2	−25	ns	1.2 ± 0.7	0.5 ± 0.3	−58	0.001
Fibrosis	1.35 ± 0.8	0.67 ± 0.5	−50	0.01	1.2 ± 0.7	0.5 ± 0.3	−60	0.001

**Table 4:** Assignments of the *m/z* ratios detected in the positive ion MALDI-TOF mass spectra of the organic extracts of serum patient NASH before (T0) and after 12 months of chronic administration of RA (T12).

*m/z* (MH^+^)	Identity	CTR	Group 1		Group 2	
			T_0_	T_12_	T_0_	T_12_
496,36	lyso PC 16:0	100	100	100	100	100
369,37	CL (−H_2_O) (H^+^)	68 ± 5	35 ± 3	16 ± 1	15 ± 7	41 ± 5
520,4	lyso PC 18:2	37 ± 3	12 ± 2	2 ± 1	11 ± 3	9 ± 4
522,41	lyso PC 18:1	30 ± 3	18 ± 5	17 ± 5	17 ± 4	15 ± 6
524,37	lyso PC 18:0	31 ± 4	32 ± 5	30 ± 3	30 ± 4	59 ± 6
703,5	SM 16:0	20 ± 5	5 ± 6	1 ± 1	8 ± 5	18 ± 2
758,65	PC 16:0/18:2	104 ± 4	21 ± 5	8 ± 2	19 ± 4	64 ± 10
760,51	PC 16:0/18:1 (H^+^)	42 ± 5	24 ± 4	13 ± 5	8 ± 2	27 ± 4
784,66	PC 18:1/18:2	43 ± 5	13 ± 3	5 ± 2	9 ± 4	34 ± 4
786,53^#^	PC 18:0/18:2 (H^+^)/ PC 18:1/18:1 (H^+^)	40 ± 6	9 ± 1	4 ± 1.5	6 ± 2	18 ± 3
804,52	PC 18:2/18:2 (Na^+^)	9 ± 1	2 ± 0.5	1 ± 0.75	2 ± 1	10 ± 0.5
808,55^#^	PC 18:0/18:2 (Na^+^)/PC 18:1/20:4 (H^+^)	19 ± 2	3 ± 0.5	9 ± 1	5 ± 1	19 ± 2
810,55	PC 18:0/20:4	25 ± 3	5 ± 1	3 ± 1	5 ± 2	18 ± 3
812,62	PC 18:0/20:3	12 ± 1	5 ± 0.5	22 ± 5	3 ± 1	16 ± 4

Quantitative determination in % made only on the basis of the value of H^+^ or Na^+^.

^#^Identification is not unique (there are two possible identities).

CL = free cholesterol; lyso-PC = lysophosphatidylcholine; PC = phosphatidylcholine; SM = sphingomyelin.

## Abbreviations

ALT: Alanine aminotransferase
AST: Aspartate aminotransferase
*γ*GT: *γ*-glutamyltransferase
BMI: Body mass index
NAFLD: Nonalcoholic fatty liver disease
NASH: Nonalcoholic steatohepatitis
HCC: Hepatocellular carcinoma
FFA: Free fatty acids
LPC: Lysophosphatidylcholine
SM: Sphingomyelin
PC: Phosphatidylcholine
ROS: Reactive oxygen species
NO: Nitric oxide
HSP27: Heat shock protein 27
TBA: Thiobarbituric acid
NAS: NAFLD activity score
PUFA: Polyunsaturated fatty acid.
